# Correction of an Error, Proved to Be No Error at All

**Published:** 1856-03

**Authors:** S. D. Gross


### EDITORIAL DEPARTMENT.

### *Correction of an Error, Proved to be no Error at all.*

By S. D. Gross, M. D.

In the *Northern Lancet* for September, 1855, the editor, Dr. Horace Nelson, in a brief notice of the second edition of my work on the Urinary Organs, has deemed it necessary to call in question the truth of certain statements in the appendix to that treatise respecting the prevalence of calculous diseases in the district of Montreal. These statements were based upon a communication kindly sent me by Professor Hall, of McGill College, by whom their correctness has been fully and triumphantly established in the February number of the *Montreal Medical Chronicle*. The paper of Dr. Hall is accompanied by the certificates of eight physicians and surgeons, residents of the district referred to, all testifying to the infrequency of calculous disorders within its limits. A brief analysis of these documents is all that will be necessary for my present purpose.

Dr. Campbell, Professor of Surgery in McGill College, asserts that, in a practice of twenty years, during nineteen of which he has been connected with the medical staff of the Montreal General Hospital, he has not seen more than a dozen cases of renal calculi, and only eight of vesical calculi; that he has performed the operation of lithotomy three times, and has witnessed it four times in the hands of his professional brethren; and, finally, that, during the last twenty-two years, there have been only three persons cut for stone in the bladder, in the Montreal General Hospital, with an annual average, during that period, of about eighty patients, and an extensive daily attendance of out-door cases at the dispensary.

The testimony of Dr. Holmes, Professor of Medicine in McGill College, perfectly coincides with that of Dr. Campbell. He avers that calculous complaints in the district of Montreal are "far from frequent," and that Dr. Hall's statements, referred to in my treatise, are "generally correct." Dr. William Belin, of L'Assomption, in a practice of nearly thirty years, has met with only three cases of urinary calculi, none of which required operation. Dr. J. B. Johnston, of Sherbrooke, has been in active practice, at that town, for fifteen years, has never seen an instance of the disease, either in his own district or in that of his professional acquaintances, extending over a circuit of thirty miles. Dr. Robert H. Wight, of St. Johns, testifies that he has practised at St. Johns and Laprairie twenty-one years, and that during all that time he had treated only two cases of vesical calculi. Dr. Francis N. Sherriff, of Huntingdon, and Dr. Charles Smallwood, of St. Martins, during a similar period, have each observed four cases of the disease.

In addition to these certificates, Profess r Hall has appealed to the statistics of the Montreal General Hospital and the Hôtel Dieu. From this it appears that, during a period of twenty years, from 1825 to 1846, not a single case of calculous disease was admitted into the former of these institutions, and only two since that period. At the Hôtel Dieu, during a similar period, only five or six cases have presented themselves.

From all these facts it is evident that calculous diseases *are* very infrequent in the district of Montreal, and that the statements in my work on the Urinary Organs, in reference to this subject, *are* true.

The attack of the editor of the *Northern Lancet* upon Professor Hall, appears to have been provoked by the fact that this gentleman, in his communication to me on the subject of calculous diseases, omitted all allusion to Dr. Robert Nelson, "who," the editor asserts, "has operated close on to, if not more than, one hundred times in less than twenty years." This seems to have been Dr. Hall's great sin, the head and frout of his offendings; but here, again, the learned and ingenious Professor is triumphant; for in the certificate of Dr. J. S. Brigham, of Philipsburg, appended to the article in the *Montreal Monthly Chronicle*, that gentleman positively affirms that he had heard Dr. Robert Nelson declare, in 1839, that he had, up to that period, performed the operation of lithotomy only thirty-nine times.

I have thus given a succinct but faithful analysis of the facts adduced by my friend, Professor Hall, in vindication of the correctness of his statements to me in regard to the subject under consideration. The reader will not fail to draw his own conclusions.

Finally, I may be permitted to state, that the facts of which the appendix in my work on the urinary organs consist, were furnished me, in every instance, by physicians of the most reliable character for truthfulness and power of observation.

